# Resveratrol suppresses neuroinflammation to alleviate mechanical allodynia by inhibiting Janus kinase 2/signal transducer and activator of transcription 3 signaling pathway in a rat model of spinal cord injury

**DOI:** 10.3389/fnmol.2023.1116679

**Published:** 2023-02-16

**Authors:** Jie Han, Zhen Hua, Wen-jie Yang, Shu Wang, Fang Yan, Jun-nan Wang, Tao Sun

**Affiliations:** ^1^Department of Pain Management, Shandong Provincial Hospital Affiliated to Shandong First Medical University, Jinan, China; ^2^College of Sports Medicines and Rehabilitation, Shandong First Medical University and Shandong Academy of Medical Sciences, Tai’an, China

**Keywords:** neuropathic pain, spinal cord injury, resveratrol, Janus kinase 2/signal transducer and activator of transcription 3, neuroinflammation

## Abstract

**Background:**

Neuropathic pain (NP) is one of intractable complications of spinal cord injury (SCI) and lacks effective treatment. Resveratrol (Res) has been shown to possess potent anti-inflammatory and anti-nociceptive effects. In this study, we investigated the analgesic effect of Res and its underlying mechanism in a rat model of SCI.

**Methods:**

The rat thoracic (T10) spinal cord contusion injury model was established, and mechanical thresholds were evaluated during an observation period of 21 days. Intrathecal administration with Res (300 μg/10 μl) was performed once a day for 7 days after the operation. On postoperative day 7, the expressions of tumor necrosis factor-α (TNF-α), interleukin-1β (IL-1β) and interleukin-6 (IL-6) were determined by enzyme-linked immunosorbent assay (ELISA) and Real-time quantitative PCR (RT-qPCR), the expression of Janus kinase 2/signal transducer and activator of transcription 3 (JAK2/STAT3) signaling pathway was determined by western blot and RT-qPCR, and the co-labeled phospho-STAT3 (p-STAT3) with neuronal nuclear antigen (NeuN), glial fibrillary acidic protein (GFAP), and ionized calcium-binding adapter molecule 1 (Iba-1) were explored by double immunofluorescence staining in the lumbar spinal dorsal horns. The temporal changes of p-STAT3 were investigated by western blot on the 1st, 3rd, 7th, 14th, and 21st days after the operation.

**Results:**

Intrathecal administration with Res for 7 successive days alleviated mechanical allodynia of rats during the observation period. Meanwhile, treatment with Res suppressed the production of pro-inflammatory factors TNF-α, IL-1β and IL-6, and inhibited the expressions of phospho-JAK2 and p-STAT3 in the lumbar spinal dorsal horns on postoperative day 7. Additionally, the protein expression of p-STAT3 was significantly increased on the 1st day following the operation and remained elevated during the next 21 days, immunofluorescence suggested that the up-regulated p-STAT3 was co-located with glial cells and neurons.

**Conclusion:**

Our current results indicated that intrathecal administration with Res effectively alleviated mechanical allodynia after SCI in rats, and its analgesic mechanism might be to suppress neuroinflammation by partly inhibiting JAK2/STAT3 signaling pathway.

## Introduction

Most individuals with traumatic spinal cord injury (SCI) suffer from different types of pain, among which neuropathic pain (NP) occurs in approximately 58% of patients (Hunt et al., 2021). This NP is severe, agonizing and unremitting (Bryce et al., 2012), which extremely impairs physical and emotional function in individuals after SCI (Kim et al., 2020). Pathogenesis of SCI-NP is complex and multifactorial (Fakhri et al., 2021). Neuroinflammation has been widely demonstrated to be crucial for the generation and maintenance of SCI-NP (Georgieva et al., 2019; Xue et al., 2022). Unfortunately, the SCI-NP is refractory to many existing pharmacological treatments, which can have serious side effects (Hatch et al., 2018). Therefore, it is essential to explore new therapeutic approaches.

Resveratrol (3,4′,5-trihydroxystilbene, Res), a natural phytoalexin polyphenol extracted from many natural plants (Tian and Liu, 2020), exerts widely beneficial health effects, including anti-inflammatory, cardiovascular protective and tumor treatment (Breuss et al., 2019; Meng et al., 2021; Ren et al., 2021), etc. Effects of Res on the relief of NP have also been extensively studied. A number of researches have shown that Res attenuates NP after peripheral nerve injury through balancing pro-inflammatory and anti-inflammatory cytokines release (Tao et al., 2016) or suppressing microglia-mediated neuroinflammation (Wang et al., 2020). In a rat model of oxaliplatin-induced NP, intrathecal administration with Res reduced the expressions of reactive oxygen species, tumor necrosis factor-α (TNF-α), interleukin-1β (IL-1β) in the spinal cord and attenuated mechanical allodynia (Dong et al., 2022). Also, treatment with Res orally decreased the serum TNF-α levels and whole brain nitric oxide release, attenuated thermal hyperalgesia in a mouse model of diabetic NP (Sharma et al., 2007). Recently, accumulating evidence suggests that Res plays a neuroprotective role through reducing neuroinflammation in nervous system diseases, such as traumatic brain injury, stroke and SCI (Xie et al., 2019; Mota et al., 2020; Zhao et al., 2021). However, literatures that the treatment of SCI with Res focus mostly on functional recovery (Liu et al., 2011; Zhao et al., 2021; He et al., 2022), and few studies have specifically investigated the central analgesic effect of Res in SCI.

The Janus kinase 2/signal transducer and activator of transcription 3 (JAK2/STAT3) signaling pathway plays a major role in many physiological and pathological processes including inflammation reaction (Morris et al., 2018). It has been reported that JAK2/STAT3 signaling pathway is involved in the neuroinflammation reaction of stroke (Zhu et al., 2021) and Alzheimer’s disease (Xue et al., 2021). Res is considered as a natural inhibitor of the STAT3 pathway (Deng et al., 2007). Recent study showed that resveratrol-selenium nanoparticles reduced the protein expression of p-STAT3 as well as IL-1β levels, and suppressed neuroinflammation in Alzheimer’s disease (Abozaid et al., 2022). Under the condition of NP induced by peripheral nerve injury, the JAK2/STAT3 signaling pathway in the lumbar spinal dorsal horns was activated and mediated interleukin-6 (IL-6) induced mechanical allodynia and thermal hyperalgesia (Dominguez et al., 2008). In addition, it was well proven that the JAK2/STAT3 signaling pathway had a rapidly activation at the injury site after SCI (Zheng et al., 2021). However, few studies have explored the expression of JAK2/STAT3 signaling pathway in the remote spinal cord after SCI, and it remains unclear whether Res can relieve SCI-NP by mediating the JAK2/STAT3 signaling pathway.

In this study, we established rat thoracic SCI model, and carried out evaluation of pain behavior and molecular changes to determine the analgesic effect and mechanism of intrathecal administration with Res for treating below-level NP after SCI.

## Materials and methods

### Animals

Male Sprague–Dawley rats (7 weeks, 260–300 g) were purchased from the Laboratory Animal College of Shandong First Medical University. All experimental rats were fed with a 12 h light/dark cycle at 25°C and had free access to rodent water and food. According to the International Association for the Study of Pain guidelines for pain research in animals, all animal experiments were approved by the Animal Care and Use Committee at the Shandong Provincial Hospital affiliated to Shandong First Medical University. Rats were distributed into sham group, SCI group, vehicle group, and Res group randomly.

### Intrathecal catheter implantation and spinal cord contusion injury

Before establishment of SCI model, polyethylene catheters (PE-10) were implanted as described for intrathecal drug administration (Choi et al., 2005). After L5–L6 intervertebral foramen was exposed, PE-10 catheters were inserted into the epidural space and gently advanced caudally to the lumbar enlargement of the spinal cord. Intrathecal catheterization was verified to be successful when the lower limbs of a rat dragged or were paralyzed by intrathecally injecting 2% lidocaine (10 μl). The internalized catheter was fixed with paravertebral muscles, and the externalized catheter was fixed firmly under the skin and sutured at the head. All rats were allowed to recover for 3 days and rats with neurological deficit, infection, or catheter prolapse were excluded from experiments.

SCI model was made using modified Allen’s method (Khan et al., 1999). In short, 2% avertin (300 mg/kg, i.p) was used for general anesthesia. The skin around the T8-T11 segment in the back was disinfected and a longitudinal incision was made. The tendons and muscle tissue were separated to expose the T10 spinous processes and lamina, and the T10 lamina was then removed, which exposes the corresponding spinal cord. A 10 g, 2.0 mm diameter rod was vertically released from a height of 30 mm through a glass tube onto the exposed spinal cord. Sham-operated animals underwent the same laminectomy procedure without contusion injury. After SCI or sham operation, the hemostatic suture was performed using 3–0 silk thread, and antibiotics were then injected subcutaneously. Rats were intramuscularly injected with 20 × 10^4^ U/d penicillin for 5 days and received artificial micturition twice daily until self-voiding was resumed.

### Drug administration

Resveratrol (Sigma-Aldrich, United States) was dissolved in 100% dimethyl sulfoxide (DMSO, Med Chem Express, China). With a 25-μl microinjection syringe connected to the intrathecal catheter, the administration Res (300 μg) or vehicle (DMSO) was performed in a volume of 10 μl over a period of 2 min, followed by 5 μl of DMSO for flushing. Drugs and vehicles were injected for 7 successive days since the first day after operation. The doses of Res were selected according to a previous study (Bermúdez-Ocaña et al., 2006; Yin et al., 2013) and our preliminary experiments.

### Mechanical hypersensitivity

Assessment of 50% paw withdrawal threshold (PWT) to mechanical stimulus was performed to evaluate the pain-related behavior, as described previously (Chaplan et al., 1994). The investigator was blinded to the medication of rats. Rats (*n* = 8/group) were habituated to the testing environment for 30 min before the behavioral test. Tests were performed on 1st day before the operation and on the 7th, 10th, 14th, and 21st after the operation.

A total of eight von Frey hairs (0.4, 0.6, 1, 2, 4, 6, 8, and 15.0 g, Stoelting, United States) were used to measure 50% PWT in rat hind paws following the “up-down” method. The filament of 2 g was used first. Then, the intensity of the next filament was decreased when the animal reacted or increased when the animal did not respond. Withdrawal of claws, shaking or licking were considered painful reactions. When the response change was observed for the first time, this procedure was continued for six stimuli. 50% PWT was calculated using the following formula: 50% PWT = 10^(Xf + κδ)^ (Xf is the logarithm value of the last von Frey fiber, and K is the corresponding value of the sequence, *δ* = 0.224). Bilateral rat hind paws were tested. Finally, the average of 50% PWT of bilateral hind paws was taken.

### Tissue sample collection

The lumbar enlargement (L4-L6) of spinal dorsal horns was harvested for various analyses and all rats were sacrificed by 2% avertin anesthesia (300 mg/kg, i.p). To explore effect of Res on the expressions of target molecules, all rats in each group were sacrificed on postoperative day 7. To study the protein of p-STAT3 at different time points after the operation, the rats in the SCI group were sacrificed on the 1st, 3rd, 7th, 14th, and 21st days after the operation. Rats in the sham group were euthanized on postoperative day 21. Before tissue collection, rats were perfused fully with normal saline *via* the heart until their livers became white. All of those tissues were collected, frozen in liquid nitrogen, and stored at −80°C until further analysis. For double immunofluorescence staining, rats were perfused with freshly prepared 4% paraformaldehyde.

### Western blot analysis

Proteins from samples (*n* = 4/group) were extracted in RIPA lysis buffer (Solarbio, China) with PMSF and a phosphatase inhibitor, and the protein concentration was assessed by BCA protein assay kit (Solarbio, China). Proteins were separated on a 7.5% sodium dodecyl sulfate-polyacrylamide electrophoresis gel (SDS-PAGE) and then transferred onto polyvinylidene fluoride membranes. The membranes were blocked with 5% non-fat milk in TBST for 1 h and incubated overnight at 4°C in primary antibodies: rabbit anti-JAK2 (1:2,000; Cell Signaling Technology, United States), rabbit anti-phospho-JAK2 (Y1007 + 1,008) (1:2,000; Abcam, United States), rabbit anti-STAT3 (1:2,000; Cell Signaling Technology, United States), rabbit anti-phospho-STAT3 (Tyr705) (1:2,000; Cell Signaling Technology, United States), rabbit anti-β-actin (1:4,000; Proteintech Group, China). Later, the membranes were washed in TBST and then cultured with anti-rabbit HRP-conjugated secondary antibody (1:4,000; Proteintech Group, China) for 1 h. Finally, the enhanced chemiluminescence assay kit (Proteintech Group, China) was used to visualize immunoblots, and the densities of the relative target proteins were measured using ImageJ. The β-actin was chosen as the internal reference control.

### Enzyme-linked Immunosorbent assay

On postoperative day 7, protein levels of the inflammatory cytokines TNF-α, IL-1β and IL-6 in the spinal dorsal horns (*n* = 4/group) were detected. Samples were homogenized and centrifuged as described previously (Liu et al., 2016). After the supernatant was collected, the cytokine levels were evaluated using rat ELISA kits (TNF-α: MultiSciences, China; IL-1β: MultiSciences, China; IL-6: MultiSciences, China). Total protein concentrations were determined by BCA protein assay kit (Solarbio, China) and used to adjust results for sample size. Cytokine levels were expressed in picograms per milligram (pg/mg).

### Real-time quantitative PCR

Samples (*n* = 4/group) were collected on postoperative day 7 and total RNAs were extracted using RNAex Pro Reagent (Accurate Biotechnology, China). cDNA was synthesized using Evo M-MLV RT Mix Kit (Accurate Biotechnology, China). Polymerase chain reaction (PCR) amplifications were conducted using SYBR® Green Premix Pro Taq HS qPCR kit (Accurate Biotechnology, China). RT-qPCR was carried out using Light Cycler® 480 II (Roche, Switzerland). GAPDH was served as the internal reference for normalization. The mRNA levels of TNF-α, IL-1β, IL-6, JAK2 and STAT3 were calculated using the 2-ΔΔCT method. The sequences of primers were obtained from Accurate Biotechnology (China) and are shown in Table 1.

**Table 1:** The Primer Sequence for RT-qPCR.

Gene	Forward primer	Reverse primer
GAPDH	5’-TGATTCTACCCACGGCAAGTT-3′	5’-TGATGGGTTTCCCATTGATGA-3′
TNF-α	5’-ATGGGCTCCCTCTCATCAGT-3′	5’-GCTTGGTGGTTTGCTACGAC-3′
IL-1β	5’-GGGATGATGACGACCTGCTA-3′	5’-ACAGCACGAGGCATTTTTGT-3′
IL-6	5’-TTTCTCTCCGCAAGAGACTTCC-3′	5’-TGTGGGTGGTATCCTCTGTGA-3′
JAK2	5’-GAAGGGTGCCCAGACGAGATT-3′	5’-TGTCCCGGATTTGATCCACCC-3′
STAT3	5’-GGGCCATCCTAAGCACAAAGC-3′	5’-CTGGATCTGGGTCTTGCCACT-3′

### Double immunofluorescence staining

Rats of SCI group (*n* = 3) were deeply anesthetized with avertin and perfused transcardially with 0.9% NaCl supplemented, followed by 4% paraformaldehyde on postoperative day 7. Lumbar enlargement tissues were cut into 20 μm cryostat sections (*n* = 3/each sample) after fixation (4% paraformaldehyde for 24 h in 4°C) and sucrose gradient dehydration. Next, the sections were treated with citric acid to retrieve antigen and then blocked with 10% donkey serum for 30 min at 37°C. The sections were then incubated in the rabbit anti-phospho-STAT3 (Tyr705) (1:200; Cell Signaling Technology, United States), mouse anti-GFAP (1:500; Servicebio, China), mouse anti-Iba-1(1:500; Servicebio, China), and mouse anti-NeuN (1:100; Servicebio, China) overnight at 4°C. After the primary antibody incubation, the sections were incubated with the corresponding secondary antibodies conjugated with CY3 and FITC for 1 h in dark conditions at 37°C. Finally, an Olympus BX60 microscope (Olympus, Japan) was applied to acquire images of the specimens.

### Data analysis and statistics

GraphPad Prism 8.3.0 was used to illustrate all the data analysis. All the data were expressed as mean ± SD. Statistical analysis and multiple comparisons were performed using SPSS 25.0 software. The Kolmogorov–Smirnov test was utilized to check whether data conformed to normal distribution. For behavioral experiments, comparisons between multiple groups were conducted by one-way ANOVA and Tukey’s *post hoc* analysis. For western blot, ELISA and RT-qPCR, multiple group comparisons were carried out by one-way ANOVA and Tukey’s or Tamhane’s T2 *post hoc* analysis. *p* < 0.05 was regarded as statistically significant.

## Results

### Resveratrol alleviates mechanical allodynia induced by SCI

**Figure 1:**
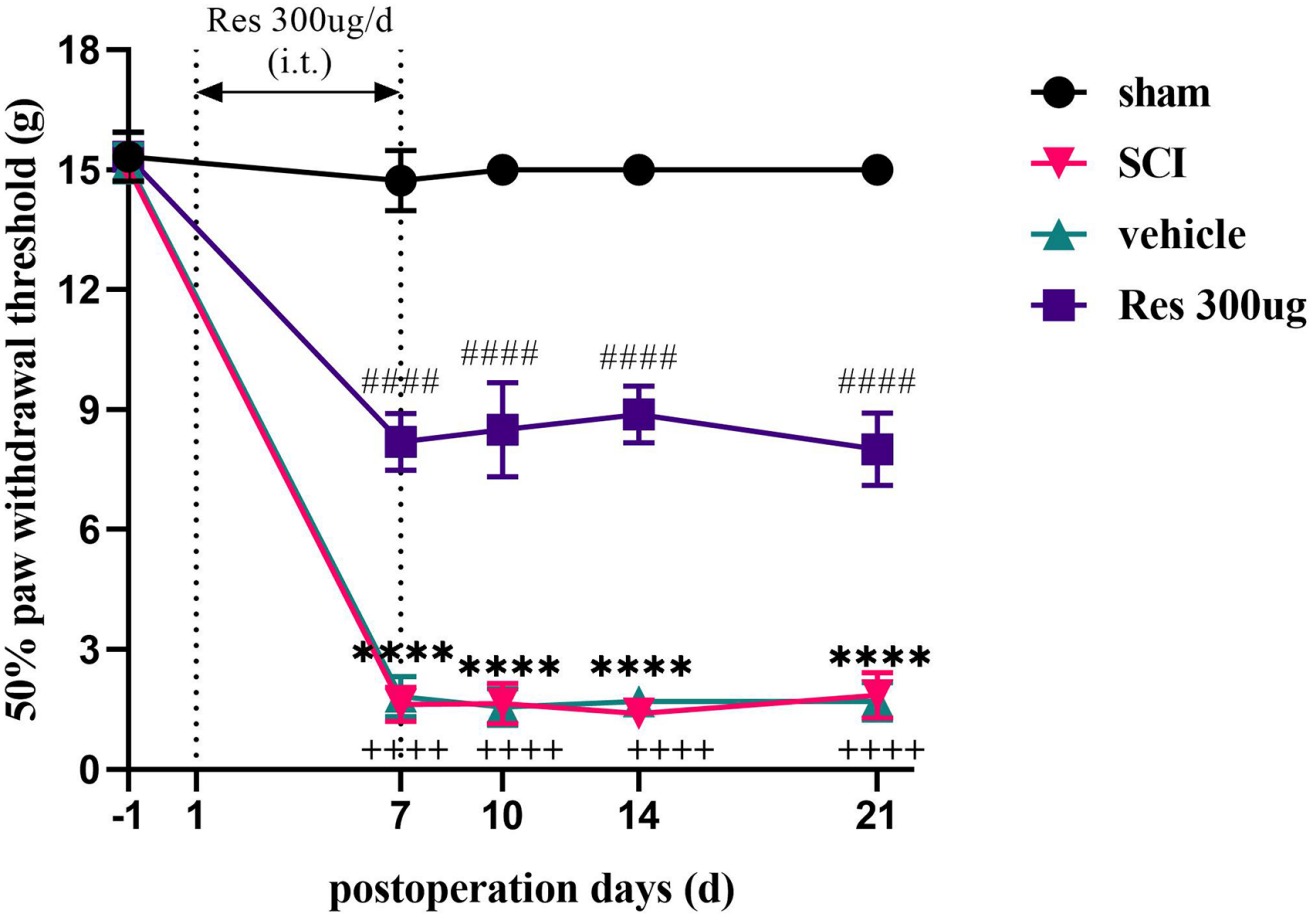
Analgesic effect of resveratrol in the model of spinal cord injury (*n* = 8/group). 50% paw withdrawal threshold was utilized to determine pain behavior in each group rats. Rats in the SCI group developed mechanical allodynia from day 7 to day 21 after the operation. Intrathecal administration with Res (300 μg/10 μl) for 7 successive days alleviated the mechanical allodynia from day 7 to day 21. All data were calculated as mean ± SD. (i.t.): intrathecal injection. ^****^p < 0.0001, the SCI group vs. the sham group; ^++++^*p* < 0.0001, the vehicle group vs. the sham group; ^####^*p* < 0.0001, the vehicle group vs. the Res group.

In order to investigate whether Res could alleviate SCI-NP, we first assessed the pain behavior of rats. As shown in Figure 1, 50% PWT was natural and normal prior to the operation in each group. Compared with the sham group, the 50% PWT in the SCI group was significantly decreased from postoperative day 7 to day 21 (*p* < 0.05), which indicated that the SCI model was established successfully. There was no significant difference in 50% PWT between the SCI group and the vehicle group at each time point after the operation (*p* > 0.05). However, compared with the vehicle group, intrathecal administration with Res for 7 successive days alleviated significantly the mechanical allodynia from day 7 to day 21 after the operation (*p* < 0.05).

### Resveratrol inhibits the expressions of phospho-JAK2 and phospho-STAT3 in the lumbar spinal dorsal horns

**Figure 2:**
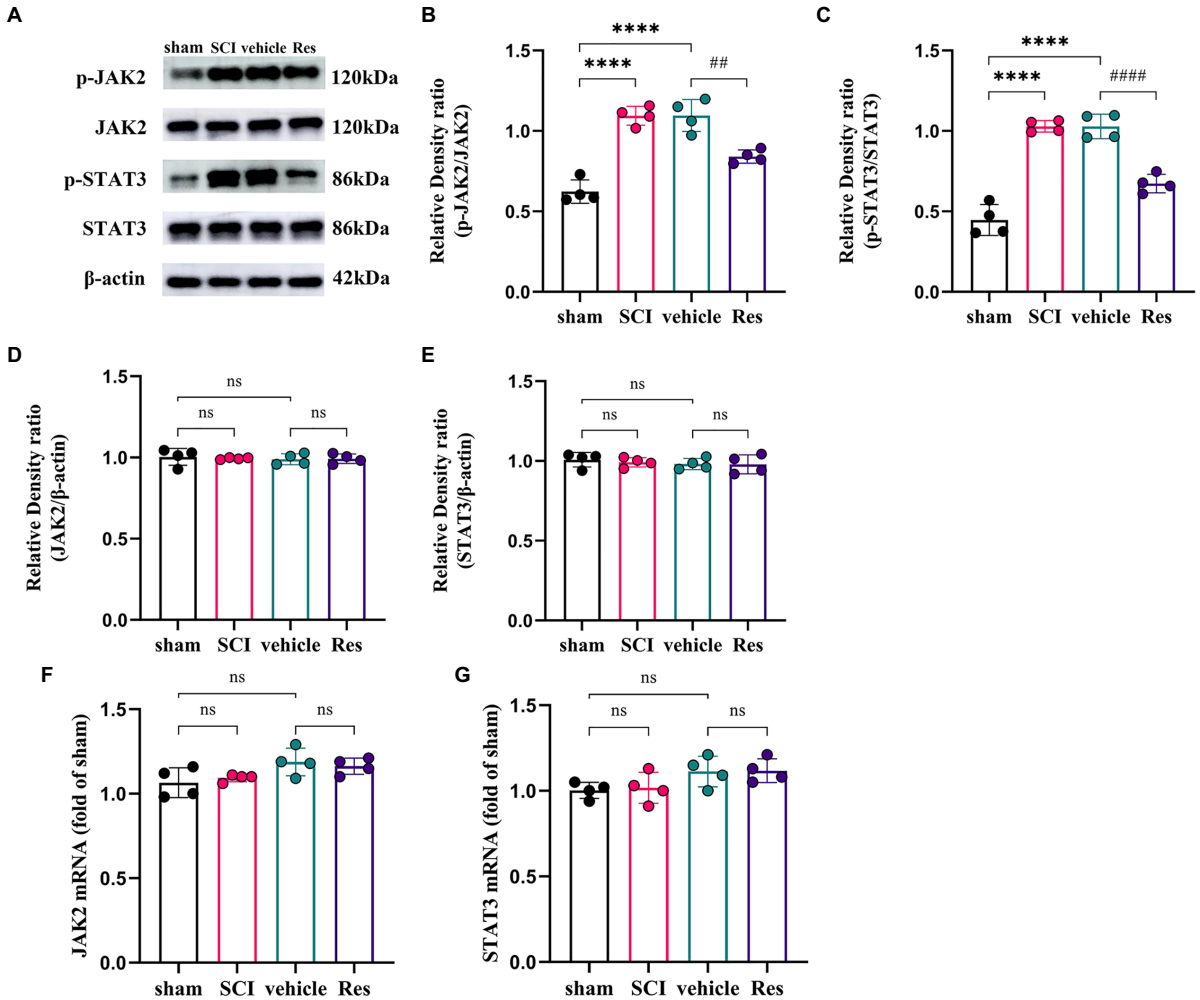
Resveratrol inhibits the protein expressions of p-JAK2 and p-STAT3 in the lumbar spinal dorsal horns (*n* = 4/group). **(A–C)** Western blot bar graphs and quantitative data of relative densities showed that the expressions of p-JAK2 and p-STAT3 were markedly increased in the SCI group on postoperative day 7. Intrathecal administration with Res (300 μg/10 μl) for 7 successive days reduced the protein levels of p-JAK2 and p-STAT3. **(D–G)** The mRNA and protein levels of JAK2 and STAT3 showed no significant differences between each group. All data were calculated as mean ± SD. ^****^p < 0.0001, ^##^*p* < 0.01, ^####^*p* < 0.0001.

To explore the potential molecular mechanism of the analgesic effect of Res in SCI-NP. The expressions of JAK2/STAT3 signaling pathway related molecules were determined by western blot and RT-qPCR in the lumbar spinal dorsal horns. On postoperative day 7, the protein levels of phospho-JAK2 (p-JAK2) (*p* < 0.05) and phospho-STAT3 (p-STAT3) (*p* < 0.05) were remarkably activated in the SCI group compared with those in the sham group. Compared with the vehicle group, intrathecal administration with Res significantly reduced the protein levels of p-JAK2 (*p* < 0.05) and p-STAT3 (*p* < 0.05) (Figures 2A–C). Interestingly, there was no significant difference in the mRNA and protein levels of JAK2 (*p* > 0.05) and STAT3 (*p* > 0.05) among the groups (Figures 2D–G).

### Resveratrol reduces the production of pro-inflammatory cytokines in the lumbar spinal dorsal horns

**Figure 3:**
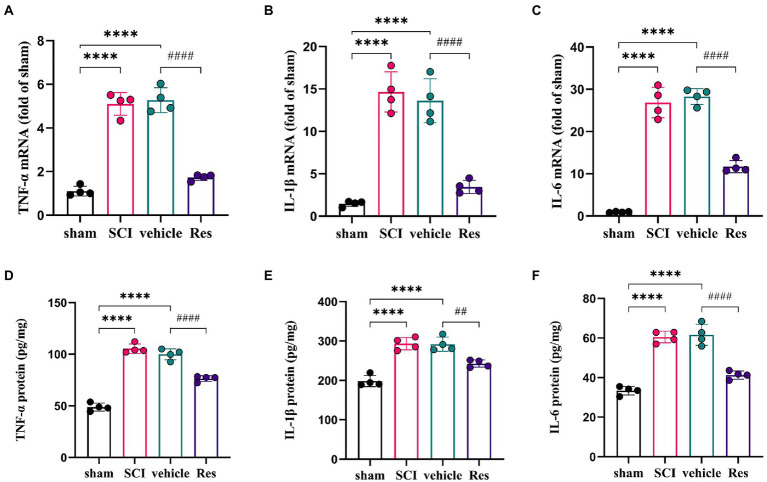
Resveratrol suppresses the inflammatory response in the lumbar spinal dorsal horns (*n* = 4/group). **(A-C)** RT-qPCR was employed to measure the mRNA levels of inflammatory cytokines. There was a significant increase in the mRNA levels of TNF-α, IL-1β and IL-6 in the SCI group and vehicle group compared with the sham group on postoperative day 7. Intrathecal administration with Res (300 μg/10 μl) for 7 successive days decreased the mRNA production of TNF-α, IL-1β and IL-6. **(D-F)** ELISA was employed to measure the protein levels of inflammatory cytokines. There was a significant expression in the protein levels of TNF-α, IL-1β and IL-6 in the SCI group and vehicle group compared with the sham group on postoperative day 7. Intrathecal administration with Res (300 μg/10 μl) for 7 successive days reduced the protein production of TNF-α, IL-1β and IL-6. All data were calculated as mean ± SD. ^****^p < 0.0001, ^##^*p* < 0.01, ^####^*p* < 0.0001.

To examine whether Res could suppress the neuroinflammation reaction. RT-qPCR and ELISA were performed to determine the levels of pro-inflammatory cytokines in the lumbar spinal dorsal horns (Figure 3). On postoperative day 7, the mRNA and protein expressions of TNF-α, IL-1β and IL-6 were markedly increased in the SCI group and vehicle group compared with the sham group (*p* < 0.05). However, compared with the vehicle group, intrathecal administration with Res decreased significantly the mRNA and protein levels of TNF-α, IL-1β and IL-6 (*p* < 0.05).

### The temporal changes and cellular localization of activated p-STAT3 in the lumbar spinal dorsal horns in SCI-NP

**Figure 4:**
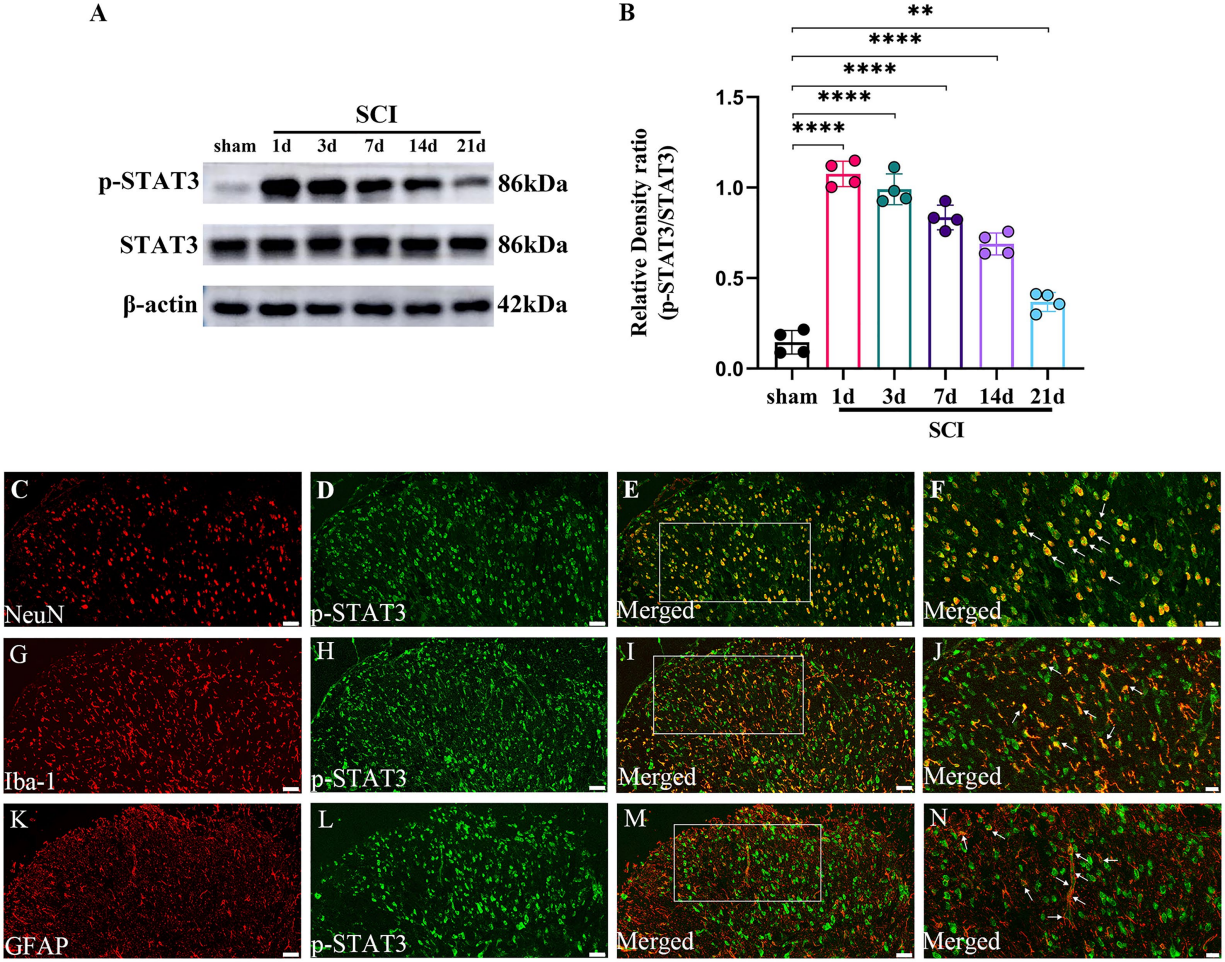
The activated p-STAT3 accumulates in the neurons and glial cells, and maintains high expression during the observation period in the lumbar spinal dorsal horns. **(A,B)** The temporal changes of activated p-STAT3 in the sham group (the day 21 after the operation) and SCI group (the day 1 to day 21 after the operation) were measured by western blot (*n* = 4/each time point). **(C–N)** The cellular localization of up-regulated p-STAT3 in the lumbar spinal dorsal horns was detected by immunohistochemistry (*n* = 3/the SCI group). Immunofluorescence staining of p-STAT3 (green) with NeuN (neuronal marker, red); GFAP (astrocyte marker, red); and Iba-1 (microglial marker, red) in the lumbar dorsal horns of SCI rats. Magnification, 200x; Scale bar, 50 μm **(C–E,G-I,K–M)**. Magnification, 400x; Scale bar, 20 μm **(F,J,N)**. All data were calculated as mean ± SD. ^**^p < 0.01, ^****^p < 0.0001.

The formation of p-STAT3 is the key to the function of JAK2/STAT3 pathway in signal regulation including inflammation (Morris et al., 2018). So, we further investigate the temporal and spatial changes of activated p-STAT3 in the lumbar spinal dorsal horns in SCI-NP. The temporal changes of p-STAT3 were determined in the sham group (the 21st day after the operation) and SCI group (the day 1 to day 21 after the operation). The results from western blot showed that the protein expression of p-STAT3 was significantly increased in the SCI group at day 1 following the operation and remained elevated during the next 21 days compared with the sham group (*p* < 0.05) (Figures 4A,B). Moreover, double immunofluorescent staining was performed to detect the cellular localization of up-regulated p-STAT3 in the lumbar spinal dorsal horns (Figures 4C–N). The results revealed that p-STAT3 was localized in the neurons, astrocytes and microglia of the lumbar spinal dorsal horns of SCI rats.

## Discussion

SCI-NP is one of the most difficult complications to treat due to the complex pathogenesis. The current drug tolerance dose cannot reliably produce satisfactory remission effect for most patients (Finnerup and Baastrup, 2012). Herein, based on the anti-inflammatory and neuroprotective effects of Res, we first discovered that intrathecal Res could alleviate mechanical allodynia of below-level NP in the thoracic SCI rat model. Moreover, treatment with Res inhibited the expressions of pro-inflammatory factors, p-JAK2, and p-STAT3, which suggested that the analgesic effect of Res depended partly on the inhibition of JAK2/STAT3 signaling pathway.

Oral Res has the characteristics of rapid absorption but low bioavailability (Walle et al., 2004). Intrathecal drug delivery has been considered as a highly favorable pharmacologic delivery system given that spinal cord dorsal contains a majority of therapeutic drug target (Karri et al., 2022). Previous research reported that intrathecal administration with Res had higher bioavailability and blood brain penetrability to treat rat intracranial glioblastomas compared to systematic administration (Shu et al., 2015). Moreover, intrathecal Res has been well studied in NP after peripheral nerve injury, however, there is no report on intrathecal Res to treat central NP after SCI (Miguel et al., 2021). So, we applied the treatment mode of intrathecal administration in this research. Res is considered as a safe and nontoxic substance (Juan et al., 2002). In the current study, we also did not observe increased mortality and abnormal behaviors in the Res group rats that received intrathecal Res at a dose of 300 μg for 7 days, which was consistent with previous studies (Bermúdez-Ocaña et al., 2006; Yin et al., 2013).

Neuroinflammation is an essential component of the pathogenesis of SCI-NP (Hulsebosch et al., 2009). The inflammatory cytokines, such as TNF-α, IL-1β and IL-6, release rapidly and perform a vital function in the generation and maintenance of SCI-NP. Previous study showed that TNF-α and IL-1β in the lumbar spinal cord began to increase at an early stage after SCI, while IL-6 increased relatively late, and all of which were negatively related to the reduction of mechanical pain threshold (Detloff et al., 2008). Compared with the “SCI no pain” group, rats of “SCI pain” group had a higher IL-6 levels (Guptarak et al., 2013), and treatment with IL-6 receptor antibody could alleviate mechanical allodynia (Guptarak et al., 2013; Murakami et al., 2013). Moreover, clinical research found that patients with SCI-NP had higher expressions of serum pro-inflammatory cytokines than those without NP (Davies et al., 2007), and targeted reduction of inflammation could improve NP symptoms in SCI (Allison et al., 2016). Therefore, targeted inhibition of inflammatory response may be an effective treatment strategy for SCI-NP.

Res is a kind of polyphenolic substance with multiple beneficial effects, which shows strong anti-inflammatory effect under various pathological conditions including SCI (Fan et al., 2021; Zhao et al., 2021). Furthermore, it has been found that Res serves as a potent analgesic by inhibiting neuroinflammation in several NP models of rats (Tao et al., 2016; Wang et al., 2020; Dong et al., 2022). In the current study, the pro-inflammatory factors were significantly increased in the lumbar spinal dorsal horns after SCI, accompanied by the decrease of mechanical pain threshold in rats. After intrathecal administration with Res, the pro-inflammatory factors TNF-α, IL-1β and IL-6 levels descended significantly, and the mechanical pain of SCI rats was ameliorated. Our data indicated that Res alleviated mechanical allodynia by inhibiting the inflammation reaction of the lumbar spinal dorsal horns in rats.

However, the potential mechanism of Res in anti-inflammatory and analgesic effect is unclear yet. The JAK2/STAT3 signaling pathway exerts an important role on the inflammation reaction (Bousoik and Montazeri Aliabadi, 2018) and may be involved. As a member of non-receptor tyrosine kinase, JAK2 is inactive prior to cytokine exposure. However, binding of cytokine to its receptor induces their auto-activation by transphosphorylation, then, the p-JAK2 can continue to induce STAT3 phosphorylation, and the p-STAT3 enters the nucleus to regulate various cellular reactions (Feng et al., 1997). For example, IL-6, a classical upstream cytokine of JAK2/STAT3 signaling pathway, can bind to its receptors and activate JAK2 and STAT3 phosphorylation, the p-STAT3 translocates to the nucleus where its binding to DNA increases cytokine gene expression to generate more interleukins including TNF-α, IL-1β and IL-6 (Satriotomo et al., 2006). It was found that the expressions of p-JAK2 and p-STAT3 increased at the injured site and mediated the transformation of microglia and astrocytes into neurotoxic phenotype, thus promoting the inflammatory response after SCI (Wang et al., 2021; Zhang et al., 2021). Numerous studies have proved that the activation of microglia and astrocyte is essential for development and maintenance of SCI-NP (Detloff et al., 2008; Chen et al., 2014). Targeted inhibition of p-STAT3 could reduce the expressions of inflammatory factors TNF-α and IL-6, inhibit apoptosis, and promote neuronal differentiation and functional recovery after SCI (Wu et al., 2019; Li et al., 2021). Recently, JAK2 inhibitor was reported to exert therapeutic effects by interfering with numerous pro-inflammatory cytokines *via* inhibition of the JAK2/STAT3 signaling pathway in COVID-19 patients (La Rosée et al., 2020). Additionally, studies have indicated that JAK2/STAT3 signaling pathway was associated with the development of NP (Dominguez et al., 2008; Ding et al., 2018; Hu et al., 2020; Zhao et al., 2022). Hu et al. (2020) found that the activated p-STAT3 at the peripheral nerve injury site mediated the increase of IL-6 in the dorsal root ganglion by retrograde transportation, and then IL-6 induced microglia activation by activating STAT3 phosphorylation, thereby promoting pain signal transmission from peripheral to central. Res is also found to play an anti-inflammatory role in a variety of pathological states by regulating JAK2/STAT3 signaling pathway. For example, Res inhibited vascular inflammation to improve symptoms of patients with rheumatoid arthritis by inhibiting STAT3 signal (Nazari-Khanamiri and Ghasemnejad-Berenji, 2022). In the current study, we found that the expressions of p-JAK2 and p-STAT3 were markedly increased in lumbar spinal dorsal horns after thoracic SCI, while intrathecal administration with Res significantly decreased the expressions of p-JAK2 and p-STAT3. In addition, we mainly focused on the expression of the terminal effector molecule p-STAT3 in the JAK2/STAT3 signaling pathway. The same method as the previous articles was adopted (Dominguez et al., 2008; Xue et al., 2022). Considering the injury such as bleeding 1 day after the operation in the sham group, we selected the rats in the sham group on the 21st day after the operation as the control. Moreover, previous study showed that the expression of p-STAT3 in the injured site increased immediately after SCI and gradually decreased within 7 days, but it was still significantly higher than that in the control group (Yamauchi et al., 2006), which partly reminded us that the comparison between the SCI group and sham group was more convincing at terminal point of the observation period. The results from immunofluorescence showed that the activated p-STAT3 was expressed in glial cells and neurons. These results indicated that the up-regulated p-STAT3 could contribute to the release of pro-inflammatory factors in glial and neurons. Therefore, we speculated that the analgesic effect of Res might be connected with the reduction of pro-inflammatory cytokines by partly inhibiting the JAK2/STAT3 signaling pathway. Of note, in the NP model of peripheral nerve injury, the activation of p-STAT3 in the spinal dorsal horn is mainly located in microglia (Dominguez et al., 2008; Hu et al., 2020), which suggests that there is a difference in the pathological mechanism between the NP of peripheral nerve injury and SCI-NP.

Previous studies mainly investigated the treatment with Res for the functional recovery of SCI, but few studies paid attention to the SCI-NP. We demonstrated the central analgesic effect of intrathecal Res and preliminary explored its molecular mechanism for the first time in SCI-NP. Our study has some limitations, the therapeutic dose of Res under different pathological conditions is not uniform, we did not further explore the effect of dose change on mechanical allodynia. Additionally, compared with systematic administration (Zhao et al., 2021; Jiang et al., 2022), the intrathecal administration with Res did not observe significant improvement in motor function, and there was no difference in motor function between groups, which requires further study in the future.

## Conclusion

In conclusion, our findings showed that intrathecal administration with Res could reduce inflammation to alleviate mechanical allodynia possibly by inhibiting JAK2/STAT3 signaling pathway in rats with SCI. This discovery confirms the central analgesic effect of Res after SCI, which might provide a new promising therapeutic strategy for the clinical treatment of SCI-NP and expand the application of Res in the field of pain.

## Data availability statement

The original contributions presented in the study are publicly available. This data can be found here: https://www.jianguoyun.com/p/DXxYyfUQoKSdCxiGoesEIAA.

## Ethics statement

The animal study was reviewed and approved by the Animal Care and Use Committee at the Shandong Provincial Hospital affiliated to Shandong First Medical University.

## Author contributions

JH and ZH carried out the major part of the study for making SCI model, detecting molecular indicators, the statistical analyses, and writing the manuscript. W-jY helped to conduct behavioral tests. SW performed the part of the western blot study. FY performed the part of statistical analyses. TS and J-nW designed the study and revised the manuscript. All authors contributed to the article and approved the submitted version.

## Funding

This article was supported by the grants from the National Natural Science Foundation of China (grant nos. 81772443 and 81972145) and Natural Science Foundation of Shandong Province (grant no. ZR2020MH283).

## Conflict of interest

The authors declare that the research was conducted in the absence of any commercial or financial relationships that could be construed as a potential conflict of interest.

## Publisher’s note

All claims expressed in this article are solely those of the authors and do not necessarily represent those of their affiliated organizations, or those of the publisher, the editors and the reviewers. Any product that may be evaluated in this article, or claim that may be made by its manufacturer, is not guaranteed or endorsed by the publisher.
